# A National Survey of Pediatric Emergency Medicine Clinicians’ Comfort Level, Beliefs, and Experiences With Initiating Buprenorphine in the Emergency Department

**DOI:** 10.7759/cureus.69331

**Published:** 2024-09-13

**Authors:** Dan Rittenhouse, Stephen Sandelich

**Affiliations:** 1 Pediatrics, Penn State College of Medicine, Hershey, USA; 2 Pediatric Emergency Medicine, Penn State Health Milton S. Hershey Medical Center, Hershey, USA

**Keywords:** buprenorphine, medication-assisted treatment, medications for opioid use disorder (moud), opioid use disorders, pediatric emergency department

## Abstract

Introduction: The prevalence of opioid use disorder (OUD) among adolescents has seen an alarming rise, prompting an exploration of the utilization of buprenorphine as a treatment modality. This study aimed to understand the perceptions, experiences, and comfort levels of pediatric emergency medicine (PEM) clinicians in initiating medication-assisted treatment (MAT) for pediatric patients presenting with OUD in emergency departments.

Methods: Utilizing a cross-sectional survey design, data from 110 respondents were collected from a potential participant pool of 3062 for a response rate of 3.6%. The survey assessed demographics, clinical encounters, comfort levels, and opinions concerning the use of buprenorphine in the pediatric emergency setting.

Results: Only 3.6% of respondents frequently evaluated and treated opioid withdrawal in the ED. A significant majority, 87.3%, indicated they had never prescribed buprenorphine for patients under 18 in the ED. While 53% of clinicians believed in initiating buprenorphine for adolescents with OUD in the ED, 33.6% expressed feeling "very uncomfortable" with the initiation process. Training in MAT appeared to influence attitudes and comfort levels significantly.

Conclusions: In this convenience sample survey with a 3.6% response rate, we found that a pronounced discomfort exists among PEM clinicians in initiating MAT, predominantly rooted in a lack of experience. Addressing this barrier through comprehensive training, developing evidence-based protocols, and fostering interdisciplinary collaboration is imperative to ensure optimal care for adolescents with OUD in emergency settings.

## Introduction

Opioid use disorder (OUD) is an intensifying public health concern, with the pediatric demographic affected at increasing rates. Data from 2020 reveals that approximately 396,000 adolescents between 12 and 17 years old engaged in opioid misuse in the United States, with about 80,000 diagnosed with OUD within that year [1]. From 1999 to 2016, the mortality rate due to OUD in the pediatric group increased by 268%, and by 2016, 12% of fatalities among individuals aged 15-24 were attributable to opioids in the United States [2-4].

Buprenorphine, a partial agonist at the mu receptor, has been proven effective in treating OUD and preventing overdose deaths in adults and adolescents [5]. In 2003, the U.S. Food and Drug Administration (FDA) approved the use of buprenorphine for patients 16 and older to treat opioid use disorder [6]. Despite its confirmed safety and extensive evidence supporting its efficacy, barriers exist that keep emergency department physicians from routinely prescribing buprenorphine for OUD patients [7,8]. Previously, a notable obstacle was the mandatory Drug Enforcement Administration (DEA) X-waiver that physicians were required to obtain before prescribing, which involved an eight-hour X-waiver training module and caps on the number of patients a clinician could treat with buprenorphine. Substance Abuse and Mental Health Services Administration (SAMHSA) data indicated that in 2017, less than 4% of licensed physicians had an x-waiver to prescribe buprenorphine [9]. To address this potential barrier, Congress passed the Consolidated Appropriations Act in 2023, which removed the requirement for an X-waiver to prescribe buprenorphine [10]. However, new or renewing DEA registrants (other than those certified in addiction medicine or addiction psychiatry) must attest to having completed a total of eight hours of training related to opioid or other substance use disorders [10].

Despite the substantial evidence advocating the initiation of buprenorphine as a secure and efficient practice, a treatment disparity persists in the pediatric cohort. Adolescents are less frequently prescribed medications for opioid use disorder (MOUD) than adults [11]. In one study, only 2.4% of adolescents treated for heroin addiction received medication-assisted treatment (MAT) compared to 26.3% of adults [12]. Moreover, only about 1% of all patients presenting to the pediatric emergency department (ED) for opioid overdose were prescribed buprenorphine within a month [13]. In 2020, the total annual rate of youth aged 19 years of age or less who had at least one buprenorphine prescription filled was 4.17 individuals per 100,000, which dropped by 45% from 2015 [14]. The rate of buprenorphine prescriptions dispensed to youth aged 19 and younger in the emergency department decreased from 3.1 to 2.3 prescriptions per 1000 persons from 2015 to 2020, a 55% decrease [14]. The reasons for these findings remain underinvestigated.

Pediatric patients with OUD presenting to the emergency department for any reason represent a critical opportunity to initiate MAT [15]. About 5% of adolescents report that the ED is their only source of medical care, meaning the ED may be the only opportunity to address and treat substance use in a portion of this population [15]. Given that many adults with OUD first encountered opioid misuse during adolescence, early interventions may mitigate the risk of further complications, including fatalities [16,17]. Compared to those who received only behavioral health services, adolescents and young adults with OUD who received buprenorphine were 42% less likely to discontinue any addiction treatment and 27% less likely to discontinue behavioral health services [18]. Case studies of adolescent patients being treated with buprenorphine in the pediatric emergency department have shown positive results, but substantial data regarding its use in pediatric emergency settings and any associated barriers to initiation are lacking [19,20]. 

A study published in 2021 explored emergency medicine residents' attitudes, experiences, and preferences on initiating buprenorphine in the emergency department [21]. However, no such research has gauged the insights of pediatric emergency medicine (PEM) clinicians. This study aims to explore their comprehension of opioid misuse and comfort in introducing MAT for OUD in pediatric patients visiting the ED. Findings from this study could highlight potential barriers to pediatric access to buprenorphine and physicians' reluctance to prescribe these medications in the ED.

This article was previously presented as a poster at the 2024 ESPR Annual Meeting on March 10, 2024 [22].

## Materials and methods

This survey study employed a cross-sectional, descriptive approach. Study data were collected and managed using REDCap electronic data capture tools [23,24]. REDCap (Research Electronic Data Capture) (Harvard Catalyst, Boston, MA) is a secure, web-based software platform designed to support data capture for research studies, providing an intuitive interface for validated data capture, audit trails for tracking data manipulation and export procedures, automated export procedures for seamless data downloads to common statistical packages, and procedures for data integration and interoperability with external sources. The Human Research Protection Program (HRPP)/Institutional Review Board approved the study on May 30, 2023. The survey was distributed via the Brown Pediatric Emergency Medicine listserv. The list encompasses attending physicians, PA/NPs, pharmacists, and trainees in PEM. Following the survey's debut in June 2023, reminders were dispatched weekly for three weeks. The survey concluded in August 2023.

The survey consisted of 14 questions. Participation was voluntary, and partially completed surveys were also included. The survey gauged participants’ roles, demographic details, experiences, comfort levels, and perceptions regarding buprenorphine treatment for OUD in the pediatric emergency setting. The survey questions can be seen in Table 1.

**Table 1:** Survey

Survey	
What is your role in your emergency department?	☐Physician ☐Resident ☐Student ☐PA/NP ☐Pharmacist ☐Other
Do you work in a community or an academic emergency department?	☐Community ☐Academic
What country do you practice in?	­___________
How many years have you been working in emergency medicine?	☐In training ☐0-5 ☐5-10 ☐10-15 ☐>15
How often do you evaluate and treat patients in your emergency department for opiate withdrawal?	☐Never ☐Infrequently ☐Often ☐Every Shift
How much do you agree with this statement?: Buprenorphine should be initiated in the emergency department for adolescents presenting with opioid-use disorder (OUD).	☐Strongly disagree ☐Somewhat disagree ☐Neither agree nor disagree ☐Somewhat agree ☐Strongly agree
How much do you agree with this statement?: Buprenorphine is effective in treating OUD and withdrawal symptoms in adolescent patients in the emergency department.	☐Strongly disagree ☐Somewhat disagree ☐Neither agree nor disagree ☐Somewhat agree ☐Strongly agree
Have you ever had training regarding medication-assisted treatment for OUD?	☐Yes ☐No
Have you ever prescribed buprenorphine in the emergency department to an adolescent patient (<18 years)?	☐Yes ☐No
How comfortable are you with initiating medication-assisted treatment for OUD?	☐Very uncomfortable ☐Uncomfortable ☐Somewhat uncomfortable ☐Somewhat comfortable ☐Comfortable ☐Very comfortable
What aspect of medication-assisted treatment for OUD in the pediatric population, if any, makes you uneasy or uncomfortable?	☐Lack of experience ☐Medication dosing ☐Initiating the conversation ☐Lack of evidence ☐Concern for diversion and misuse ☐Not applicable
I estimate that ___ (percentage) of my pediatric emergency colleagues are comfortable using buprenorphine for treatment for OUD.	☐<10% ☐25% ☐50% ☐75% ☐100% ☐unsure
How much do you agree with this statement?: My emergency room has a clear protocol in place for initiating medication-assisted therapy for opiate withdrawal.	☐Strongly disagree ☐Somewhat disagree ☐Neither agree nor disagree ☐Somewhat agree ☐Strongly agree
How important is it for a pediatric emergency medicine provider to be comfortable with medication-assisted treatment?	☐No opinion ☐Not important at all ☐Somewhat important ☐Important ☐Very important

Statistical analysis was executed using SAS software (version 9.4, SAS Institute Inc., Cary, NC). Descriptive statistics were generated via redcap, including means, medians, and standard deviations for continuous variables, which were presented as frequencies and percentages. Subgroup evaluations were conducted based on position, setting, experience duration, training in MAT, previous experience prescribing buprenorphine, and frequency of evaluating opioid withdrawal in the ED. These risk differences between groups were analyzed with 95% confidence intervals. Group variances on categorical outcomes were assessed using Pearson’s chi-square statistic or Fisher’s exact test.

## Results

Out of the 3062 potential participants, 110 responded to the survey, giving a response rate of 3.6% of the total members. Demographics are presented in Table 2.

**Table 2:** Demographics

Role	N=110
Emergency medicine attending physician	12 (10.9%)
Pediatric emergency medicine attending physician	81 (73.6%)
Other	17 (15.5%)
Setting	
Community	18 (16.4%)
Academic	92 (83.6%)
Years of experience	
In-training	14 (12.7%)
0–5 years	27 (24.5%)
5–10	20 (18.2%)
10–15	19 (17.3%)
>15	30 (27.3%)

Most respondents identified as PEM attending physicians (81/110; 73.6%), with 10.9% identifying as emergency medicine attending physicians (12/110) and 15.5% as “other (17/110).” The 17 respondents identifying as “other” could not be definitively placed into a category for subgroup analyses but most likely represented PEM fellows, as there was not a “fellow” answer choice for the first question that inquired about the role. Ninety-two participants (83.6%) reported practicing in an academic emergency department, while the remaining (18/110; 16.4%) worked in a community setting. About 13% were in training (14/110), 24.5% possessed 0-5 years of experience (27/110), 18.2% had between 5-10 years (20/110), 17.3% fell within the 10-15 years bracket (19/110), and the largest segment, 27.3%, reported having over 15 years of experience (30/110).

When addressing their clinical encounters, a significant proportion (66/110; 60.0%) reported infrequent evaluations and treatments of patients for opioid withdrawal in the emergency department. Only a minuscule portion, 3.6% (4/110), frequently engaged in such assessments and treatments, and an even smaller fraction, 1.8% (2/110), reported these encounters during every shift. Notably, 34.5% (38/110) had never evaluated or treated opioid withdrawal cases in the emergency setting.

About 53% of participants either somewhat agree (40/110; 36.4%) or strongly agree (18/110; 16.4%) with the statement that buprenorphine should be initiated in the emergency department for adolescents presenting with OUD. In response to the same statement, under a third of participants (34/110; 30.9%) selected “neither agree nor disagree,” and 11.8% selected “somewhat disagree (13/110).”

When asked how much they agreed that buprenorphine is effective in treating OUD and withdrawal symptoms in adolescent patients in the emergency department, the majority of respondents (41/110; 37.3%) selected “neither agree nor disagree,” 28.2% selected “somewhat agree (31/110), and 30.0% selected “strongly agree (33/110).” Most respondents (71/110; 64.5%) reported they have never had training for medication-assisted treatment for opioid use disorder. Delving deeper into clinical practice, an overwhelming 87.3% (96/110) had never prescribed buprenorphine for patients under 18 in the ED setting.

Participants expressed varying degrees of comfort with initiating MAT in the pediatric emergency room. A majority, 33.6% (37/110), indicated being "very uncomfortable," followed by 26.4% (29/110) who were "uncomfortable." Few respondents expressed confidence, with only 5.5% (6/110) indicating they were "comfortable" and 7.3% (8/110) stating they were "very comfortable." When assessing peer perspectives, the predominant belief (84.5%; 93/110) was that fewer than 10% of their PEM colleagues were comfortable using buprenorphine for OUD treatment.

Most respondents (69/110; 62.7%) stated that their emergency department did not have a clear protocol for initiating medication-assisted therapy for opioid withdrawal in adolescents. 

Among respondents, lack of experience was the most frequently cited source of discomfort with prescribing buprenorphine (93/110; 84.5%). Table 3 shows the full list of sources of discomfort along with the frequency each selection.

**Table 3:** Sources of discomfort

Source of discomfort	N=110
Lack of experience	93 (84.5%)
Medication dosing	46 (41.8%)
Initiating the conversation	16 (14.5%)
Lack of evidence	6 (5.5%)
Concern for diversion and misuse	20 (18.2%)
Difficulty finding adequate follow-up	46 (41.8%)
None	3 (2.7%)

Notably, among PEM physicians, a substantial 87.7% (71/81) had never prescribed buprenorphine to adolescent patients in the ED. For PEM physicians, lack of experience was a significant source of uneasiness towards initiating buprenorphine for teenage patients, with 85.2% identifying this as an area of discomfort. Lack of evidence was not a substantial worry among PEM physicians, as 95.1% (77/81) did not identify this as a concern.

There were no significant differences in the responses based on the clinician's role in the emergency department and practice setting. There was a statistically significant difference in the groups based on years of experience and medication dosing as a source of discomfort (Table 4). Among participants in training or who completed their training within the last five years, 63.4% selected medication dosing as an area of discomfort with MAT. Only 35.9% of those with 5-15 years of experience and 20.0% of those in practice over 15 years selected this as a source of uneasiness with initiating buprenorphine (p=0.0008).

**Table 4:** Subgroup analysis based on the question: "How long have you been working in emergency medicine?" CI: confidence interval. *Chi-square test used. ^+^Fisher's exact test used.

		In training/0–5 years (total N = 41)		5–15 years (total N = 39)		>15 years (total N = 30)		P-value
		Percent (N)	95% CI	Percent (N)	95% CI	Percent (N)	95% CI	
Agree with: “Buprenorphine should be initiated in the emergency department for adolescents with OUD”	Strongly/somewhat disagree	9.8% (4)	0.5–19.0	12.8% (5)	2.2–23.5	26.7% (8)	11.1–44.1	0.2^*^
	Neither agree nor disagree	39.0% (16)	23.9–54.2	25.6% (10)	11.7–39.6	26.7% (8)	11.1–44.1	
	Somewhat/strongly agree	51.2% (21)	35.7–66.8	64.1% (25)	46.0–77.1	43.3% (13)	26.4–63.2	
Agree with: “Buprenorphine is effective in treating adolescents with OUD.”	Strongly/somewhat disagree	2.4% (1)	0.0–7.4	2.6% (1)	0.0–7.6	6.67% (2)	0.0–15.7	0.6^+^
	Neither agree nor disagree	41.5% (17)	26.9–58.1	41.0% (16)	25.3–56.7	26.7% (8)	10.6–42.7	
	Somewhat/strongly agree	53.7% (22)	39.3–70.7	56.4% (22)	40.6–72.2	66.7% (20)	49.5–83.8	
How comfortable are you initiating MAT for OUD?	Very uncomfortable/uncomfortable	70.7% (29)	56.6–84.9	53.8% (21)	38.0–69.7	53.3% (16)	35.2–71.5	0.1^+^
	Somewhat uncomfortable/comfortable	26.8% (11)	13.1–40.6	28.2% (11)	13.9–42.6	26.7% (8)	10.6–42.7	
	Comfortable/very comfortable	2.4% (1)	0.0–7.2	18.0% (7)	5.7–30.2	20.0% (6)	5.5–34.5	
Agree with: “My ED has a clear protocol in place for MAT”	Strongly/somewhat disagree	80.5% (33)	68.2–92.8	69.2% (27)	54.5–84.0	63.3% (19)	45.8–80.9	0.2^+^
	Neither agree nor disagree	9.8% (4)	0.5–19.0	5.1% (2)	0.0–12.2	10.0% (3)	0.0–20.9	
	Somewhat/strongly agree	9.8% (4)	0.5–19.0	25.6% (10)	11.7–39.6	50.0% (15)	10.6–42.7	
How important is it for a PEM (clinician) to be comfortable with MAT?	Not important at all/somewhat important	63.4% (26)	50.0–80.0	41.0% (16)	27.0–59.5	46.7% (14)	32.7–71.0	0.2^*^
	Important/very important	34.1% (14)	20.0–50.0	53.8% (21)	40.5–73.0	43.3% (13)	29.0–67.3	
Sources of discomfort	Lack of experience	95.1% (39)	88.4–100.0	79.5% (31)	66.6–92.4	76.7% (23)	61.3–92.0	0.06^*^
	Medication dosing	63.4% (26)	48.4–78.4	35.9% (14)	20.6–51.2	20.0% (6)	5.5–34.5	0.0008^*^
	Initiating the conversation	12.2% (5)	2.0–22.4	20.5% (8)	7.6–33.4	10.0% (3)	0.0–20.9	0.4^*^
	Lack of evidence	12.2% (5)	2.0–22.4	2.6% (1)	0.0–7.6	0.0% (0)	0.0–0.0	0.07^+^
	Concern for misuse/diversion	22.0% (9)	9.1–34.8	17.9% (7)	5.7–30.2	13.3% (4)	1.0–25.7	0.06^*^
	Difficulty with follow-up	39.0% (16)	23.9–54.2	56.4% (22)	40.6–72.2	26.7% (8)	10.6–42.7	0.04^*^

The participants who responded that they never or infrequently treated OUD were significantly less comfortable initiating MAT than those who treated OUD often or every shift (p<0.001) (Table 5). Additionally, those who treated OUD often or every shift more often felt that it was important for PEM physicians to be comfortable treating OUD with MAT. In regards to sources of discomfort with treating OUD, those who never or infrequently treated OUD were more likely to have discomfort with lack of experience (p=0.046), medication dosing (p=0.04), and difficulty with follow-up (p=0.004).

**Table 5:** Subgroup analysis based on the question: "How often do you evaluate and treat patients in your emergency department for opiate withdrawal?" CI: confidence interval. ^+^Fisher's exact test used.

		Never/infrequently (total N = 104)		Often/every shift (total N = 6)		P-value
		Percent (N)	95% CI	Percent (N)	95% CI	
Agree with: “Buprenorphine should be initiated in the emergency department for adolescents with OUD”	Strongly/somewhat disagree	15.4% (16)	8.4–22.6	16.7% (1)	0.0–47.0	0.2^+^
	Neither agree nor disagree	32.7% (34)	23.8–42.2	0.0% (0)	0.0–0.0	
	Somewhat/strongly agree	51.0% (53)	41.7–61.3	83.3% (5)	53.0–100.0	
Agree with: “Buprenorphine is effective in treating adolescents with OUD”	Strongly/somewhat disagree	3.8% (4)	0.1–7.7	0.0% (0)	(0.0–0.0)	0.1^+^
	Neither agree nor disagree	39.4% (41)	30.2–49.4	0.0% (0)	(0.0–0.0)	
	Somewhat/strongly agree	55.8% (58)	46.6–66.0	100.0% (6)	100.0–100.0	
How comfortable are you initiating MAT for OUD?	Very uncomfortable/uncomfortable	63.5% (66)	54.1–72.9	0.0% (0)	0.0–0.0	<0.0001^+^
	Somewhat uncomfortable/comfortable	27.9% (29)	19.1–36.6	16.7% (1)	0.0–47.0	
	Comfortable/very comfortable	8.7% (9)	3.2–14.1	83.3% (5)	53.0–100.0	
Agree with: “My ED has a clear protocol in place for MAT”	Strongly/somewhat disagree	74.0% (77)	65.5–82.6	33.3% (2)	0.0–71.7	0.06^+^
	Neither agree nor disagree	7.7% (8)	2.5–12.9	16.7% (1)	0.0–47.0	
	Somewhat/strongly agree	18.3% (19)	10.7–25.8	50.0% (3)	9.4–90.6	
How important is it for a PEM (clinician) to be comfortable with MAT?	Not important at all/somewhat important	53.8% (56)	47.2–67.1	0.0% (0)	0.0–0.0	0.008^+^
	Important/very important	40.4% (42)	32.9–52.8	100.0% (6)	100.0–100.0	
Sources of discomfort	Lack of experience	86.5% (90)	79.9–93.2	50.0% (3)	9.4–90.6	0.05^+^
	Medication dosing	44.2% (46)	34.5–53.9	0.0% (0)	0.0–0.0	0.04^+^
	Initiating the conversation	15.4% (16)	8.3–22.4	0.0% (0)	0.0–0.0	0.6^+^
	Lack of evidence	5.8% (6)	1.2–10.3	0.0% (0)	0.0–0.0	1.0^+^
	Concern for misuse/diversion	18.3% (19)	10.7–25.8	16.7% (1)	0.0–47.0	1.0^+^
	Difficulty with follow-up	38.5% (40)	29.0–48.0	100.0% (6)	100.0–100.0	0.004^+^

Attending physicians and other clinicians with formal training in MAT were more likely to agree that buprenorphine should be initiated in the emergency department (p=0.007) and that buprenorphine effectively treats adolescents with OUD (p=0.04) (Table 6). They were also more comfortable starting MAT (p<0.0001), more likely to work in emergency departments with a clear protocol in place for initiating MAT (p=0.005), and felt that it was important for PEM physicians to be comfortable with starting MAT (p=0.0005). Additionally, they were less likely to list lack of experience (p=0.006) or concern for misuse or diversion (p=0.04) as aspects of MAT that made them uncomfortable.

**Table 6:** Subgroup analysis based on the question: "Have you ever had training regarding medication-assisted treatment for opiate-use disorder?" CI: confidence interval. *Chi-square test used. ^+^Fisher's exact test used.

		No training (total N = 71)		Yes, had training (total N = 39)		P-value
		Percent (N)	95% CI	Percent (N)	95% CI	
Agree with: “Buprenorphine should be initiated in the emergency department for adolescents with OUD”	Strongly/somewhat disagree	19.7% (14)	10.3–29.1	7.7% (3)	0.0–16.6	0.007^*^
	Neither agree nor disagree	38.0% (27)	26.6–49.5	17.9% (7)	5.9–30.9	
	Somewhat/strongly agree	42.3% (30)	30.6–53.9	71.8% (28)	59.5–87.9	
Agree with: “Buprenorphine is effective in treating adolescents with OUD”	Strongly/somewhat disagree	4.2% (3)	0.0–9.1	2.6% (1)	0.0–7.6	0.04^+^
	Neither agree nor disagree	45.1% (32)	33.9–57.6	23.1% (9)	9.6–36.5	
	Somewhat/strongly agree	49.3% (35)	38.1–61.9	74.4% (29)	60.4–88.3	
How comfortable are you initiating MAT for OUD?	Very uncomfortable/uncomfortable	77.5% (55)	67.6–87.3	28.2% (11)	13.9–42.6	<0.0001^*^
	Somewhat uncomfortable/comfortable	19.7% (14)	10.3–29.1	41.0% (16)	25.3–56.7	
	Comfortable/very comfortable	2.8% (2)	0.0–6.7	30.8% (12)	16.1–45.5	
Agree with: “My ED has a clear protocol in the place for MAT”	Strongly/somewhat disagree	87.3% (62)	79.5–95.2	43.6% (17)	27.8–59.4	<0.0001^*^
	Neither agree nor disagree	8.5% (6)	1.9–15.0	7.7% (3)	0.0–16.2	
	Somewhat/strongly agree	4.2% (3)	0.0–9.0	48.7% (19)	32.8–64.7	
How important is a PEM (clinician) to be comfortable with MAT?	Not important at all/somewhat important	62.0% (44)	55.1–78.2	30.8% (12)	16.6–46.6	0.0005^*^
	Important/very important	31.0% (22)	21.8–44.9	66.7% (26)	53.4–83.0	
Sources of discomfort	Lack of experience	91.5% (65)	85.0–98.1	71.8% (28)	57.5–86.1	0.006^*^
	Medication dosing	46.5% (33)	34.7–58.3	33.3% (13)	18.3–48.4	0.2^*^
	Initiating the conversation	18.3% (13)	9.2–27.5	7.7% (3)	0.0–16.2	0.1^*^
	Lack of evidence	2.8% (2)	0.0–6.7	10.3% (4)	0.6–19.9	0.2^+^
	Concern for misuse/diversion	23.9% (17)	13.9–34.0	7.7% (3)	0.0–16.2	0.03^*^
	Difficulty with follow-up	35.2% (25)	23.9–46.5	53.8% (21)	38.0–69.7	0.06^*^

The subgroup analysis of participants who have or have not been prescribed buprenorphine before revealed a significant difference in whether or not respondents agreed that buprenorphine should be initiated in the pediatric emergency department (p=0.009) (Table 7). The group that reported having prescribed buprenorphine in the past was more comfortable with buprenorphine (p=0.03) and felt that PEM physicians should be comfortable using it (p=0.01). There were no statistically significant differences in the sources of discomfort in this group.

**Table 7:** Subgroup analysis based on the question: "Have you ever prescribed buprenorphine in the emergency department to an adolescent patient (<18 years) CI: confidence interval. *Chi-square test used. ^+^Fisher's exact test used.

		Never prescribed buprenorphine (total N = 96)		Previously prescribed buprenorphine (total N = 14)		P-value
		Percent (N)	95% CI	Percent (N)	95% CI	
Agree with: “Buprenorphine should be initiated in the emergency department for adolescents with OUD”	Strongly/somewhat disagree	14.6% (14)	7.5–22.0	21.4% (3)	0.0–43.3	0.009^+^
	Neither agree nor disagree	35.4% (34)	26.0–45.6	0.0% (0)	0.0–0.0	
	Somewhat/strongly agree	49.0% (47)	39.3–59.7	78.6% (11)	56.7–100.0	
Agree with: “Buprenorphine is effective in treating adolescents with OUD”	Strongly/somewhat disagree	3.1% (3)	0.0–6.7	7.1% (1)	0.0–20.9	0.08^+^
	Neither agree nor disagree	40.6% (39)	31.0–51.1	14.3% (2)	0.0–32.9	
	Somewhat/strongly agree	55.2% (53)	45.6–65.9	78.6% (11)	56.7–100.0	
How comfortable are you initiating MAT for OUD?	Very uncomfortable/uncomfortable	64.6% (62)	54.9–74.3	28.6% (4)	4.5–52.6	0.03^+^
	Somewhat uncomfortable/comfortable	24.0% (23)	15.3–32.6	50.0% (7)	23.4–76.6	
	Comfortable/very comfortable	11.5% (11)	5.0–17.9	21.4% (3)	0.0–43.3	
Agree with: “My ED has a clear protocol in place for MAT”	Strongly/somewhat disagree	76.0% (73)	67.4–84.7	42.9% (6)	16.5–69.2	0.009^+^
	Neither agree nor disagree	8.3% (8)	2.7–14.0	7.1% (1)	0.0–20.9	
	Somewhat/strongly agree	15.6% (15)	8.3–23.0	50.0% (7)	23.4–76.6	
How important is it for a PEM (clinician) to be comfortable with MAT?	Not important at all/Somewhat important	55.2% (53)	48.6–69.2	21.4% (3)	0.0–43.3	0.01^*^
	Important/very important	38.5% (37)	30.8–51.5	78.6% (11)	56.7–100.0	
Sources of discomfort	Lack of experience	86.5% (83)	79.5–93.4	71.4% (10)	47.4–95.5	0.2^+^
	Medication dosing	44.8% (43)	34.7–54.9	21.4% (3)	0.0–43.3	0.1^*^
	Initiating the conversation	12.5% (12)	5.8–19.2	28.6% (4)	4.5–52.6	0.1^+^
	Lack of evidence	6.3% (6)	1.3–11.2	0.0% (0)	0.0–0.0	1.0^+^
	Concern for misuse/diversion	19.8% (19)	11.7–27.9	7.1% (1)	0.0–20.9	0.5^+^
	Difficulty with follow-up	40.6% (39)	30.6–50.6	50.0% (7)	23.4–76.6	0.5^*^

## Discussion

This study's findings reveal a complex interplay of factors that affect the initiation of MAT, specifically buprenorphine, in the pediatric emergency department setting. Buprenorphine's effectiveness in treating OUD and its benefits in preventing opioid overdose fatalities are well documented, particularly within the adult population [25]. However, despite its recognized efficacy and safety profile, a significant disparity exists in its prescription practices for adolescents [11-13].

One of the most striking observations from the study is the reported discomfort among PEM physicians and other clinicians with initiating MAT. This discomfort predominantly stems from a lack of experience, which is concerning given that early intervention is crucial in managing OUD and can potentially change an adolescent's life trajectory. The lack of experience and knowledge suggests a potential gap in pediatric emergency medicine fellowship training, emergency medicine, and pediatrics residency education. This highlights the importance of robust training modules that address the nuances of treating pediatric patients with OUD. 

Our findings support increased training to address the discrepancy in buprenorphine initiation in the adolescent and adult populations. Individuals who received training for MAT therapy were less likely to feel uncomfortable initiating buprenorphine. Previous training for MAT therapy was also associated with a more favorable attitude toward buprenorphine initiation. Those with previous training were more likely to agree that buprenorphine should be started in the ED for adolescent patients with OUD. They were more likely to feel that it is essential for PEM physicians and other clinicians to be comfortable with MAT therapy. Attitude can influence behavior, so this association between training and more favorable feelings towards buprenorphine therapy further indicates that the expansion of MAT training could be pivotal in increasing the initiation of buprenorphine for OUD in adolescents.

Additionally, the need for more precise protocols in community and academic emergency department settings adds another layer of complexity. This study reveals a lack of standardized guidelines for buprenorphine therapy in the pediatric emergency department. Notably, those who felt their ED lacked a clear protocol were less likely to have prescribed buprenorphine. Individual physicians may face uncertainties in clinical decision-making, which could lead to some patients receiving optimal treatments while others do not.

Moreover, it is notable that most participants, irrespective of their setting, have not prescribed buprenorphine for patients under 18. As mentioned, most PEM clinicians participating in this survey reported discomfort in initiating MAT, likely contributing to decreased initiation of buprenorphine in adolescents. Reduced initiation of MAT further reinforces the most commonly identified source of discomfort in the study: lack of experience. Clinician discomfort with initiating MAT appears to stem from other sources, including concerns about medication dosing, starting conversations about OUD, concern for diversion, and difficulty finding adequate follow-up. Further investigation would be warranted to understand the magnitude of the impact of these other factors in the hesitancy to initiate buprenorphine.

Addressing these barriers requires a multifaceted approach. Comprehensive training programs tailored to the unique challenges of pediatric OUD could equip pediatric emergency medicine physicians and other clinicians with the knowledge and confidence to initiate MAT. Moreover, interdisciplinary collaboration involving pediatric emergency physicians, advanced practice clinicians, addiction specialists, and pediatricians can aid in developing and refining evidence-based protocols, ensuring that they are both clinically sound and practical to implement.

Our study was limited by a small sample size of only 110 participants with an overall response rate of 3.6%. However, despite a relatively small number of participants, statistical significance was achieved with multiple groups in the subgroup analysis. There is a concern for selection bias as the respondents self-selected out of all recipients on the listserv. This can affect the external validity of the survey as the self-selected individuals may not be a nationally representative sample of pediatric emergency medicine physicians and other clinicians treating children and adolescents in the emergency setting. There is also the possibility of a nonresponse bias with a low response rate. Respondents who were more interested or invested in the topic may have been more likely to respond and may be biased toward positive attitudes regarding the use of MAT in the emergency department.

While we found significant differences in individuals who had previous training for MAT, the survey did not allow us to uncover differences in the groups based on the quality or quantity of their MAT training. We cannot say what kind of training or how much training is most effective in helping physicians and other clinicians become more comfortable initiating buprenorphine. However, any training appears to be better than none. Another possible limitation would be that several participants selected “other” as their role in the pediatric emergency department. Some of these participants may have been PEM fellows, but we could not include them in our subgroup analyses without knowing the precise roles of individuals in this group.

## Conclusions

In this convenience sample survey with a 3.6% response rate, we found that a significant level of discomfort exists among PEM clinicians in initiating MAT, predominantly rooted in a lack of experience. This study underscores the urgent need for systemic changes in how pediatric patients with OUD are managed in emergency departments. The findings shed light on critical barriers that hinder the optimal care of these vulnerable patients. As the medical community strives to translate research into clinical practice, policy, and education, these insights offer a roadmap for tangible improvements in pediatric emergency care that emphasize the overarching goal of ensuring the well-being and future of our adolescent population.

## Figures and Tables

**Figure 1:**
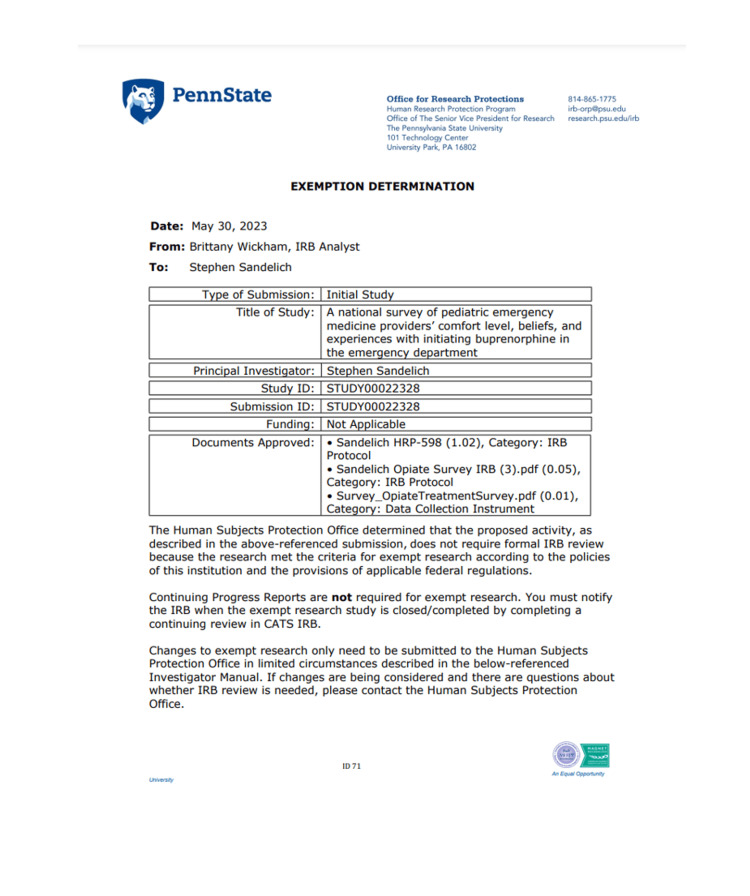
IRB exemption letter
